# Clinical Usefulness of the VS Classification System Using Magnifying Endoscopy with Blue Laser Imaging for Early Gastric Cancer

**DOI:** 10.1155/2017/3649705

**Published:** 2017-05-15

**Authors:** Yoshikazu Yoshifuku, Yoji Sanomura, Shiro Oka, Kazutaka Kuroki, Mio Kurihara, Takeshi Mizumoto, Yuji Urabe, Toru Hiyama, Shinji Tanaka, Kazuaki Chayama

**Affiliations:** ^1^Department of Gastroenterology and Metabolism, Graduate School of Biomedical Sciences, Hiroshima University, Hiroshima, Japan; ^2^Department of Endoscopy, Hiroshima University Hospital, Hiroshima, Japan; ^3^Health Service Center, Hiroshima University, Higashihiroshima, Japan

## Abstract

**Background:**

Blue laser imaging (BLI) enables the acquisition of more information from tumors' surfaces compared with white light imaging. Few reports confirm the validity of magnifying endoscopy (ME) with BLI (ME-BLI) for early gastric cancer (EGC). We aimed to assess the detailed endoscopic findings from EGCs using ME-BLI.

**Methods:**

We enrolled 386 consecutive patients with 417 EGCs that were diagnosed using ME-BLI and resected by endoscopic submucosal dissection. Using the VS classification system, three highly experienced endoscopists (HEEs) and three less experienced endoscopists (LEEs) evaluated the demarcation line (DL), microsurface pattern (MSP), and microvascular pattern (MVP) within the endoscopic images of EGCs obtained using ME-BLI, assigning high-confidence (HC) or low-confidence (LC) levels. We investigated the clinicopathological features associated with each confidence level.

**Results:**

The HEEs' evaluations determined the presence of DL in 99%, irregular MSP in 96%, and irregular MVP in 96%, and the LEEs' evaluations determined the presence of DL in 98%, irregular MSP in 95%, and irregular MVP in 95% of the EGCs. When DL was present, HC levels in the *Helicobacter pylori-* (*H. pylori-*) eradicated group and noneradicated group were evident in 65% and 89%, a difference that was significant (*p* < 0.001).

**Conclusions:**

In the diagnosis of EGC with ME-BLI, the VS classification system with ME-NBI can be applied, but identifying the DL after *H. pylori* was difficult.

## 1. Introduction

Gastric cancer is one of the most common cancers, and it is the second leading cause of cancer deaths worldwide [1]. Endoscopic submucosal dissection (ESD) is performed worldwide to treat early gastric cancer (EGC). In Japan, ESD for EGC is performed in accordance with the Japanese gastric cancer treatment guidelines [2], and it can attain higher en bloc resection and curative resection rates and good prognoses, even for large or ulcerated lesions [3–7]. To perform ESD, it is important to accurately diagnose the gastric cancer at an early stage. However, it is difficult to diagnose EGCs using white light imaging (WLI) only because EGCs can sometimes appear to be assimilated within the surrounding mucosa. Although chromoendoscopy using indigo carmine dye or an acetic acid-indigo carmine dye mixture has been reported to be useful for EGC detection [8–13], these procedures take time and effort.

In recent years, many image-enhanced endoscopy (IEE) techniques, including narrow band imaging (NBI), flexible spectral imaging color enhancement (FICE), and blue laser imaging (BLI), have been developed to improve the visualization of the vascular and surface patterns within the surface of the mucosa, and, therefore, diagnoses. Several reports describe the value of IEE for gastric tumors [14–17]. IEE is widely used because of its simplicity. Indeed, it only requires a button to be pushed, and unlike chromoendoscopy, a dye solution is not needed. Furthermore, more detailed information about EGCs can be obtained when magnifying endoscopy (ME) is combined with IEE. NBI is the form of IEE most used in Japan. Yao et al. [14] described the VS classification system, which is an approach that facilitates the diagnosis of EGC using ME and NBI, and they emphasized three factors to consider during the diagnosis of EGC, namely, the presence of a demarcation line (DL), an irregular microvascular pattern (MVP), and an irregular microsurface pattern (MSP). The criteria that define a diagnosis of EGC are either the presence of an irregular MVP with a DL or the presence of an irregular MSP with a DL.

BLI is an IEE technique that uses the LASEREO system, which is a laser-based endoscopy system. Only one publication suggests that ME-BLI may be useful for diagnosing EGC [17], and there are no reports that detail the endoscopic findings attained using ME-BLI. In the present study, we aimed to assess the validity of the VS classification system using ME-BLI for EGC.

## 1. Patients and Methods

### 1.1. Patients

We enrolled 386 consecutive patients who had 417 EGCs that were diagnosed using BLI and who had undergone ESD resections at Hiroshima University Hospital between August 2011 and March 2016.  Table 1 presents the characteristics of the study participants and their tumors. All of the patients provided written informed consent to undergo ESD. The study was approved by Hiroshima University's Institutional Review Board and its Ethics Committee. In addition, this study was performed in accordance with the principles of the Declaration of Helsinki.

### 1.2. Imaging Techniques Using the BLI System

The LASEREO endoscopy system (Fujifilm Co., Tokyo, Japan) consists of an LL-4450 light source, a VP-4450HD video processor, and any of a special series of scopes. The LL-4450 light source provides illumination through two different lasers that have mean wavelengths of 410 (standard deviation (SD) = 10) nm and 450 (SD = 10) nm. The 450 nm wavelength laser excites the white light phosphor and produces fluorescent light for standard observations, while the 410 nm wavelength laser is for BLI, which functions as narrow band imaging. The lighting setup offers three observation modes, namely, the BLI mode, the BLI-bright mode, and the white light mode, which can be selected by adjusting the intensity of the two lasers. The BLI mode is a combination of a strong 410 nm laser light, a weak 450 nm laser light, and a fluorescent light. The BLI-bright mode is a combination of a strong 410 nm laser light, a 450 nm laser that is stronger than the 450 nm laser used for the BLI mode, and a fluorescent light. The white light mode is a combination of a weak 410 nm laser light, a strong 450 nm laser light, and a fluorescent light.

We used BLI endoscopes (EG-L600ZW and EG-L590ZW; Fujifilm Co., Tokyo, Japan), light sources (LASEREO LL-4450; Fujifilm Co., Tokyo, Japan), and video processors (Advancia HD VP-4450HD; Fujifilm Co., Tokyo, Japan). For the BLI mode, the structure enhancement function and the color mode were set at the B8 level and level 1.

### 1.3. Image Evaluation

**Figure 1:**
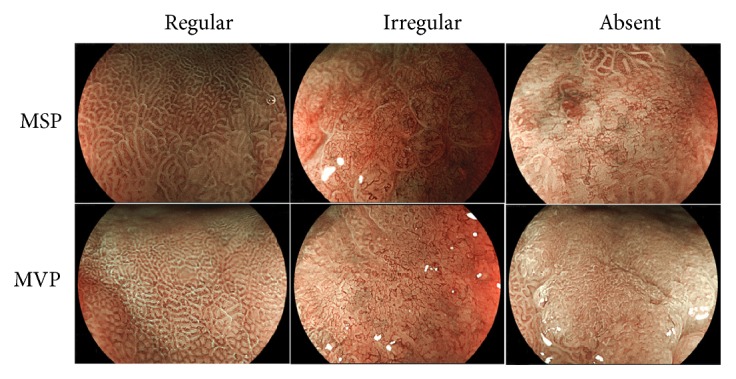
Endoscopic images of the VS classification system using ME-BLI.

Three highly experienced endoscopists (HEEs) who had diagnosed >500 cases using ME-BLI and three less experienced endoscopists (LEEs) who had diagnosed ≤500 cases using ME-BLI retrospectively participated in the evaluation of the images. The endoscopic images of the EGCs were obtained using ME-BLI at a magnification of up to ×100, and they were randomly presented to each of the physicians who evaluated the images for the presence or absence of DL and to determine whether the MSPs were regular, irregular, or absent and whether the MVPs were regular, irregular, or absent using the VS classification system (Figure 1). The endoscopists assigned high-confidence (HC) or low-confidence (LC) levels to their evaluations. If individual diagnostic interpretations differed, the three endoscopists discussed the case until consensus was reached. We investigated the clinicopathological features associated with each confidence level for the EGCs that showed the presence of DL, irregular MSP, and irregular MVP. We also assessed the interobserver agreements between the two groups of endoscopists.

### 1.4. Statistical Analysis

The quantitative data are expressed as the means and the SDs or percentages. The differences in the values were analyzed using the chi-square test with Yates' correction or using Student's *t*-test. A value of *p* < 0.05 was considered statistically significant. The interobserver agreements were measured using the kappa statistic.

## 2. Results

Table 2 presents the ME-BLI findings based on the VS classification system. The evaluation of the images by the HEEs showed that DL was present in 99% (415/417) of the EGCs and that the total HC rate was 85% (351/417). Regular, irregular, and absent MSPs were observed in 1% (6/417), 96% (401/417), and 2% (10/417) of the EGCs, respectively, and the total HC rate was 85% (356/417). Regular, irregular, and absent MVPs were observed in 1% (2/417), 96% (401/417), and 1% (3/417) of the EGCs, respectively, and the total HC rate was 93% (389/417). The evaluation of the images by the LEEs showed that DL was present in 98% (410/417) of the EGCs and that the total HC rate was 82% (342/417). Regular, irregular, and absent MSPs were observed in 2% (8/417), 95% (398/417), and 3% (11/417) of the EGCs, respectively, and the total HC rate was 84% (350/417). Regular, irregular, and absent MVPs were observed in 1% (4/417), 95% (397/417), and 4% (16/417) of the EGCs, respectively, and the total HC rate was 89% (370/417). There were no significant differences between the HEEs and LEEs with respect to the endoscopic findings.

Table 3 presents the HEEs' confidence levels with respect to the ME-BLI findings relative to the clinicopathological features of the tumors. In the EGCs in which the DL was present, HC levels were evident in 65% (51/78) and 89% (300/337) of the *Helicobacter pylori-* (*H. pylori-*) eradicated group and the *H. pylori*-noneradicated group, respectively. The HC rate in the *H. pylori-*noneradicated group was significantly higher than that in the *H. pylori-*eradicated group (*p* < 0.01). In EGCs in which the MSP was irregular, the mean tumor sizes with LC and HC levels were 8 (SD = 4) mm and 15 (SD = 11) mm, and there was a significant difference between the LC and HC levels. In EGCs in which the MVP was irregular, mean tumor sizes of LC and HC levels were 8 (SD = 5) mm and 14 (SD = 10) mm, and there was a significant difference between the LC and HC levels. The interobserver agreements with respect to the DL, MSP, and MVP for the HEEs were 0.78, 0.72, and 0.76, respectively, and for the LEEs were 0.66, 0.61, and 0.65, respectively, (Table 4). The interobserver agreements were good-to-satisfactory between the groups.

## 3. Discussion

This study's findings demonstrate that ME-BLI facilitates the acquisition of detailed information about the microvascular and microsurface patterns within the mucosal surfaces of EGCs. Currently, BLI and NBI are used widely as IEE based on narrow band observation function in esophagogastroduodenoscopy. Several publications have described the reliability of ME-NBI at characterizing and delineating EGC [14, 18–21]. The findings from a study by Yamada et al. [18] showed that the sensitivity, specificity, and accuracy of ME-NBI in relation to the diagnosis of EGC were excellent at 95%, 97%, and 97%, respectively. Ezoe et al. [19] showed that the sensitivity, specificity, and accuracy of ME-NBI in relation to the diagnosis of EGC were 95.0%, 96.8%, and 96.6%, respectively. Hence, ME-NBI yielded excellent diagnostic performances with respect to accuracy, sensitivity, and specificity in both of these studies. In addition to obtaining histopathological diagnoses of EGC, it is important to use ESD to assess the lateral extents of EGCs, and ME-NBI has been reported to be very useful at assessing the lateral extents of differentiated-type EGCs [22–25]. However, it is difficult to assess the lateral extents of undifferentiated-type EGCs, even when ME-NBI is used, because of the presence of proliferative zone extensions. Horiuchi et al. [26] reported that 81.6% of EGCs could be correctly diagnosed based on the DL when ME-NBI was used. However, it seems that this accuracy is not sufficient when the lateral extents of EGCs appropriate for ESD are being assessed, and this represents a diagnostic limitation associated with optical biopsies.

Regarding the diagnostic yield for EGCs when ME-BLI is used, Dohi et al. [17] reported that compared with WLI, ME-BLI had an improved diagnostic performance. They showed that the sensitivity, specificity, and accuracy of ME-NBI in relation to the diagnosis of EGCs were excellent at 94%, 92%, and 92%, respectively, and that the diagnostic effectiveness of ME-BLI was similar to that of ME-NBI. However, the study by Dohi et al. [17] is the only one that has investigated the clinical utility of ME-BLI, and there are no publications that have described investigations into DL, MSP, and MVP, which constitute an EGC diagnosis, the confidence levels, the need for proficiency, and the clinicopathological features of EGC using ME-BLI. In this study, the presence of a DL, an irregular MSP, and an irregular MVP was observed in more than 95% of the EGCs, in both the HEE and LEE groups, and the HC levels observed for all of these characteristics were more than 80%. There were no significant differences between the HEEs and LEEs with respect to the endoscopic findings and the confidence levels, which suggests that most of the EGC diagnoses undertaken using ME-BLI can be performed easily, regardless of the level of the endoscopist's experience.

Some of the EGC diagnoses were difficult even for the HEEs, including small EGCs or EGCs after *H. pylori* eradication. *H. pylori* infection causes gastric cancer [27–30], and metachronous EGC was reduced to one-third with successful *H*. *pylori* eradication therapy after ESD for EGC [31]. Several studies' findings show that it is often difficult to diagnose EGCs after *H. pylori* eradication therapy, because of the tumors' indistinct borderlines or lack of obviously cancerous characteristics [32–34]. Furthermore, we reported that, endoscopically, gastric cancers changed to flattened and indistinct forms after *H. pylori* eradication and that nonneoplastic epithelium covered the cancerous areas [35, 36]. The findings from this study showed that the HC rates associated with DL were 65% in the *H. pylori*-eradicated group and 89% in the *H. pylori*-noneradicated group, a difference that was significant. Hence, we should be careful when we perform endoscopic surveillance to ensure that we do not miss EGC or misunderstand the range of the tumor in patients after *H. pylori* eradication.

This study has some limitations that are described next. First, this study was retrospective and it involved a review of endoscopic images. Hence, it may not reflect the real-time prospective diagnoses that occur during surveillance endoscopy. Second, this study investigated cancerous lesions only. To avoid bias, a prospective study should be performed that enrolls lesions that have not undergone pathological diagnoses. Third, the study was conducted at a single academic center in Japan; hence, the study's data may lack generalizability to gastric cancer treatment centers worldwide.

We conclude that in diagnosis of EGC with ME-BLI, the VS classification system with ME-NBI can be applied. However, it tends to be difficult to identify the DL of EGCs after *H. pylori* eradication.

## Figures and Tables

**Table 1:** Clinical characteristics of the patients (*n* = 386) and the early gastric cancers (*n* = 417).

Characteristic	
Sex ratio (male/female)	280/106
Age, years, mean (SD)	70.3 (10.4)
Tumor location, *n* (%)	
Upper	74 (18)
Middle	138 (33)
Lower	205 (49)
Macroscopic type, *n* (%)	
0-I, 0–IIa	142 (34)
0–IIb, 0–IIc	275 (66)
Tumor size, mm, mean (SD)	18.6 (13.2)
Histological type, *n* (%)	
Differentiated	383 (92)
Undifferentiated	34 (8)
Depth, *n* (%)	
T1a	377 (90)
T1b	40 (10)
*Helicobacter pylori*	
Eradicated, *n* (%)	79 (19)
Noneradicated, *n* (%)	338 (81)

SD: Standard deviation.

**Table 2:** ME-BLI findings based on the VS classification system.

Endoscopist	Confidence level	DL		MSP			MVP		
		Present, *n* (%)	Absent, *n* (%)	Regular, *n* (%)	Irregular, *n* (%)	Absent, *n* (%)	Regular, *n* (%)	Irregular, *n* (%)	Absent, *n* (%)
HEE	High	351 (85)	0 (0)	0 (0)	350 (84)	6 (1)	0 (0)	378 (91)	11 (3)
	Low	64 (14)	2 (1)	6 (1)	51 (12)	4 (1)	2 (1)	23 (6)	3 (1)
	Total	415 (99)	2 (1)	6 (1)	401 (96)	10 (2)	2 (1)	401 (96)	14 (3)
LEE	High	342 (82)	0 (0)	0 (0)	346 (83)	4 (1)	0 (0)	360 (86)	10 (2)
	Low	68 (16)	7 (1)	8 (1)	52 (12)	7 (2)	4 (1)	37 (9)	6 (1)
	Total	410 (98)	7 (1)	8 (1)	398 (95)	11 (3)	4 (1)	397 (95)	16 (4)

DL: Demarcation line; MSP: Microsurface pattern; MVP: Microvascular pattern; HEE: Highly experienced endoscopist: LEE: Less experienced endoscopist.

**Table 3:** The HEEs' confidence levels in relation to the ME-BLI findings relative to the clinicopathological features of the tumors.

Clinicopathological feature	DL present		*p* value	MSP irregular		*p* value	MVP irregular		*p* value
	Low confidence (*n* = 64)	High confidence (*n* = 351)		Low confidence (*n* = 51)	High confidence (*n* = 350)		Low confidence (*n* = 23)	High confidence (*n* = 378)	
Location, *n* (%)									
Upper	15 (20)	59 (80)		12 (17)	60 (83)		5 (7)	66 (93)	
Middle	21 (15)	117 (85)	NS	18 (12)	128 (88)	NS	7 (5)	126 (95)	NS
Lower	28 (14)	175 (86)		21 (11)	162 (89)		11 (6)	186 (94)	
Macroscopic type, *n* (%)									
0-I, 0–IIa	15 (11)	127 (89)	NS	14 (10)	120 (90)	NS	5 (4)	132 (96)	NS
0–IIb, 0–IIc	49 (18)	224 (82)		37 (14)	230 (86)		18 (7)	246 (93)	
Tumor size, mm, mean (SD)	12 (9)	15 (11)	NS	8 (4)	15 (11)	<0.01	8 (5)	14 (10)	<0.05
Histological type, *n* (%)									
Differentiated	59 (15)	323 (85)	NS	42 (11)	329 (89)	NS	20 (5)	349 (95)	NS
Undifferentiated	5 (15)	28 (85)		9 (29)	22 (71)		3 (9)	29 (91)	
Depth, *n* (%)									
T1a	60 (16)	315 (84)	NS	49 (13)	316 (87)	NS	21 (6)	343 (94)	NS
T1b	4 (10)	36 (90)		2 (4)	44 (96)		2 (5)	35 (95)	
*Helicobacter pylori* eradication, *n* (%)									
Eradicated	27 (35)	51 (65)	<0.001	15 (20)	60 (80)	NS	8 (11)	68 (89)	NS
Noneradicated	37 (11)	300 (89)		36 (11)	290 (89)		15 (5)	310 (95)	

SD: Standard deviation; DL: Demarcation line; MSP: Microsurface pattern; MVP: Microvascular pattern; NS: Not significant.

**Table 4:** Interobserver agreements for the ME-BLI findings based on the VS classification system.

ME-BLI finding	Interobserver agreement (kappa value)	
	HEE	LEE
DL	0.78	0.66
MSP	0.72	0.61
MVP	0.76	0.65

DL: Demarcation line; MSP: Microsurface pattern; MVP: Microvascular pattern; HEE: Highly experienced endoscopist; LEE: Less experienced endoscopist.
